# Chronic intermittent hypoxia promotes glomerular hyperfiltration and potentiates hypoxia-evoked decreases in renal perfusion and PO_2_

**DOI:** 10.3389/fphys.2023.1235289

**Published:** 2023-07-06

**Authors:** Kiefer W. Kious, Kalie A. Savage, Stephanie C. E. Twohey, Aubrey F. Highum, Andrew Philipose, Hugo S. Díaz, Rodrigo Del Rio, James A. Lang, Sarah C. Clayton, Noah J. Marcus

**Affiliations:** ^1^ Department of Physiology and Pharmacology, Des Moines University Medicine and Health Sciences, Des Moines, IA, United States; ^2^ Department of Biology, Simpson College, Indianola, IA, United States; ^3^ Laboratory of Cardiorespiratory Control, Department of Physiology, Pontificia Universidad Católica de Chile, Santiago, Chile; ^4^ Facultad de Ciencias de la Salud, Instituto de Ciencias Biomédicas, Universidad Autónoma de Chile, Santiago, Chile; ^5^ Centro de Excelencia en Biomedicina de Magallanes (CEBIMA), Universidad de Magallanes, Punta Arenas, Chile; ^6^ Department of Kinesiology, Iowa State University, Ames, IA, United States

**Keywords:** sleep apnea, chemoreflex, GFR, renal blood flow, renal PO_2_

## Abstract

**Introduction:** Sleep apnea (SA) is highly prevalent in patients with chronic kidney disease and may contribute to the development and/or progression of this condition. Previous studies suggest that dysregulation of renal hemodynamics and oxygen flux may play a key role in this process. The present study sought to determine how chronic intermittent hypoxia (CIH) associated with SA affects regulation of renal artery blood flow (RBF), renal microcirculatory perfusion (RP), glomerular filtration rate (GFR), and cortical and medullary tissue PO_2_ as well as expression of genes that could contribute to renal injury. We hypothesized that normoxic RBF and tissue PO_2_ would be reduced after CIH, but that GFR would be increased relative to baseline, and that RBF, RP, and tissue PO_2_ would be decreased to a greater extent in CIH vs. sham during exposure to intermittent asphyxia (IA, F_i_O_2_ 0.10/F_i_CO_2_ 0.03). Additionally, we hypothesized that gene programs promoting oxidative stress and fibrosis would be activated by CIH in renal tissue.

**Methods:** All physiological variables were measured at baseline (F_i_O_2_ 0.21) and during exposure to 10 episodes of IA (excluding GFR).

**Results:** GFR was higher in CIH-conditioned vs. sham (*p* < 0.05), whereas normoxic RBF and renal tissue PO_2_ were significantly lower in CIH vs. sham (*p* < 0.05). Reductions in RBF, RP, and renal tissue PO_2_ during IA occurred in both groups but to a greater extent in CIH (*p* < 0.05). Pro-oxidative and pro-fibrotic gene programs were activated in renal tissue from CIH but not sham.

**Conclusion:** CIH adversely affects renal hemodynamic regulation and oxygen flux during both normoxia and IA and results in changes in renal tissue gene expression.

## Introduction

Sleep apnea (SA) is observed in 50%–60% of patients with chronic kidney disease (CKD) and is associated with increased risk of cardiovascular events, end stage renal disease (ESRD), and mortality (Huang et al., 2017; Centers for Disease Control and Prevention, 2021). In support of the notion that SA is injurious to renal health, treatment of SA with continuous positive airway pressure or adaptive servo ventilation results in improvements in renal function (Kinebuchi et al., 2004; Owada et al., 2013; Puckrin et al., 2015; Li et al., 2019). Because of these established clinical relationships, the pathophysiology underlying sleep apnea and renal dysfunction is an area of significant research interest.

Studies in SA patients have shown persistent increases in sympathetic nerve activity (SNA) (Maier et al., 2022), decreased renal plasma flow (Kinebuchi et al., 2004), glomerular hyperfiltration (Kinebuchi et al., 2004), increased activity of the renin angiotensin aldosterone system (RAAS) (Di Murro et al., 2010; Zalucky et al., 2015), as well as systemic inflammation (Unnikrishnan et al., 2015; Perrini et al., 2017; Orrù et al., 2020) and evidence of oxidative stress (Orrù et al., 2020; Hu et al., 2022). Previous studies have shown that the chronic intermittent hypoxia (CIH) associated with SA results in tonic increases in afferent carotid body chemoreflex (CBC) activity (Peng et al., 2003; Rey et al., 2004; Roy et al., 2018; Prabhakar et al., 2023) as well as changes in central chemoreflex sensitivity, both of which contribute to elevated efferent SNA (Prabhakar et al., 2023; Ostrowski et al., 2023; Gilmartin et al., 2010; Huang et al., 2009; Marcus et al., 2010; Molkov et al., 2011). Acute activation of the CBC with hypoxia has previously been shown to increase renal SNA and decrease renal blood flow (RBF) in healthy animals (Huang et al., 2009; Marcus et al., 2015; Pügge et al., 2016; Kious et al., 2022), an effect exacerbated by conditions with enhanced chemoreceptor sensitivity (Huang et al., 2009; Marcus et al., 2015; Pügge et al., 2016; Kious et al., 2022). Recent studies have also suggested that tonic increases in chemoreflex activation in the absence of hypoxic stimulation can exert a significant influence on resting normoxic RBF (Marcus et al., 2015; Kious et al., 2022). The potential clinical significance of these findings should not be underestimated given the aforementioned high prevalence of co-morbid SA in patients with CKD or ESRD. CKD patients with high chemosensitivity and SA might suffer chronic CBC-mediated increases in renal SNA and reductions in RBF which are then compounded by further reductions following apneas or hypopneas. This represents a potentially important link between sleep apnea and renal dysfunction as chemoreflex-mediated increases in renal SNA would be expected to result in chronic reductions in RBF and renal tissue oxygenation (Lin et al., 2020).

A recent study found that CIH conditioning causes reduced renal cortical oxygen tension under normoxic conditions even in the absence of significant reductions in RBF (O’Neill et al., 2019). It is important that these studies be extended to identify how CIH affects renal hemodynamic regulation and oxygen flux during repeated bouts of asphyxia as this is analogous to what a person with SA experiences at night when asleep. The importance of altered hemodynamics and decreased tissue PO_2_ is underscored by the fact that tissue hypoxia is theorized to be an important driver of renal inflammation, oxidative stress, fibrosis, and subsequent declines in kidney function (Wang et al., 2022).

Therefore, we sought to assess the effect of CIH on RBF, renal microcirculatory perfusion (RP), and cortical and medullary PO_2_ (CPO_2_, MPO_2_) at baseline and during repeated bouts of chemoreflex stimulation with intermittent asphyxia (IA). Because of the potential influence of glomerular hyperfiltration and additional solute load on renal oxygen consumption, we also did experiments to determine the effects of CIH on glomerular filtration rate (GFR) measured in conscious animals after conditioning with CIH. Finally, we explored changes in renal tissue gene expression that could link CIH conditioning, hemodynamic dysregulation, and tissue hypoxia with renal injury and fibrosis, potentially leading to reductions in renal function and development of CKD.

We hypothesized that reductions in RBF, RP, and CPO_2_/MPO_2_ during IA would be exacerbated in rats previously conditioned with CIH, and that GFR measured in un-anesthetized animals would be increased after CIH. Furthermore, we also hypothesized that CIH would result in activation of pro-oxidative pro-fibrotic gene programs. Our results indicate that CIH may facilitate development of renal dysfunction through exaggerated reduction in RBF, RP, CPO_2_, and MPO_2_ during episodes of asphyxia characteristic of SA. We also observed changes in renal tissue gene expression with CIH conditioning that may contribute to renal damage.

## Materials and methods

### Ethical approval and statement of compliance

The experimental protocols were approved by the Des Moines University Medicine and Health Sciences Institutional Animal Care and Use Committee and were conducted in accordance with the National Institutes of Health (NIH Publication No. 85-23, revised 1996) and the American Physiological Society’s Guide for the Care and Use of Laboratory Animals. The authors wish to declare that our work complies with the animal ethics checklist outlined by Grundy (Grundy, 2015).

### Experimental groups

**FIGURE 1:**
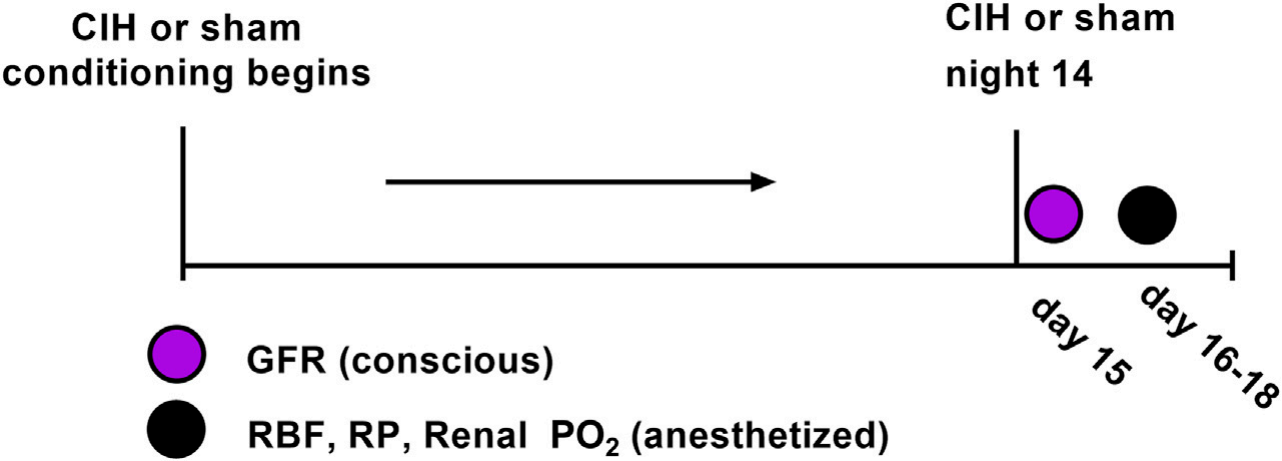
Experimental timeline.

Adult male Sprague-Dawley rats (Envigo, Madison, WI) of similar age (8–10 weeks) and weight (250–300 g) were used for these experiments. All rats were group-housed under controlled temperature (23°C) and humidity conditions and kept on a 12:12 h light-dark cycle and fed standard rat chow with water available *ad libitum*. At the beginning of the study 30 animals were randomly assigned to the following groups (*n* = 15 per group): Sham (animals exposed to air-air cycling), CIH (animals exposed to intermittent hypoxia-air cycling). In certain cases we were unable to use a given data stream or in other cases not all animals were used for all experiments. For each experimental variable, the n values are given in the results section as well as the figure legends. Both groups followed identical experimental timelines (shown in Figure 1).

### Chronic intermittent hypoxia and sham conditioning

Both experimental groups were exposed to 14 sequential days of chronic intermittent hypoxia (CIH, 60 s F_i_O_2_ 0.10, 120 s F_i_O_2_ 0.21) or normoxia/normoxia cycling (sham, 60 s F_i_O_2_ 0.21, 120 s F_i_O_2_ 0.21), as previously described (Marcus et al., 2009; Marcus et al., 2010; Philippi et al., 2010; Marcus et al., 2012). The CIH protocol ran for 8 h per day (from 07:30-15:30) and took place in a pair of adjacent Plexiglas chambers (Coy Laboratory Products Inc., Glass Lake, MI) in a designated room in the animal care facility. All animals remained in their home cages which were placed in the Plexiglas chambers during daily exposures.

### Measurement of glomerular filtration rate

At the conclusion of the CIH or sham protocol, GFR was assessed non-invasively in conscious, unrestrained rats by measuring clearance of the fluorescent tracer FITC-sinistrin as described previously (Schock-Kusch et al., 2009; Schock-Kusch et al., 2011; Friedemann et al., 2016; Herrera Pérez et al., 2016; Kious et al., 2022). Briefly, FITC-sinistrin clearance was measured using the preclinical transdermal GFR monitor (Medibeacon, St. Louis, MO). On the day prior to the study, a site on the back was shaved and prepared with a depilatory agent (i.e. Nair). On the day of the study the rats were briefly anesthetized (1.5% Isoflurane in air) and the depilated area was cleaned. The monitor was affixed to the back with an adhesive patch and a bolus injection of FITC-sinistrin (5 mg/100 g BW) (Herrera Pérez et al., 2016) was given through one of the lateral tail veins. Measurements of FITC-sinistrin fluorescence were made once per second for 120 min. At the conclusion of the experiment, the monitor and battery pack were removed, and the data was downloaded for later analysis with MPD Lab software (Medibeacon, St Louis, MO) as described previously (Friedemann et al., 2016).

### Anesthetic protocol and monitoring procedures

Experiments to measure RBF, RVC, RP, CPO_2_, and MPO_2_ were conducted 24 h after GFR measurements. For surgical physiological experiments, anesthesia was induced with 4% inhalation isoflurane in air after which time anesthesia was reduced to 1.5%–2.0% as necessary to maintain a surgical plane of anesthesia. In order to determine appropriate anesthetic depth was reached, a toe pinch and/or tail pinch was administered after induction and every 15 min thereafter. The absence of paw withdrawal, movement of the tail, vocalization, or marked increase in respirations after a pinch was taken as evidence of adequate anesthetic depth. Body temperature was measured using a rectal thermometer and monitored using a Physiosuite w/RightTemp Temperature Monitoring and Homeothermic Control system (Kent Scientific, Torrington, CT), and was maintained at 37°C using a far infrared heating pad integrated with a Surgisuite surgical platform (Kent Scientific, Torrington, CT).

### Measurement of renal blood flow, perfusion, and tissue PO_2_

After appropriate anesthetic depth was reached, the left femoral artery was cannulated with a Millar Mikro-Cath pressure catheter (Millar, Houston, TX) connected to a bridge amp (AD Instruments, Colorado Springs, CO) for measurement of arterial pressure. Then a ventral mid-line incision was made, and the kidney was approached in the peritoneal space. Once the renal artery and vein were identified the renal artery was gently separated from the vein and a transit-time flow probe (Transonic, Ithaca, NY) was placed around the renal artery. After the flow probe was secured, two needle probes were inserted 0.5–1 mm (cortical) and 3–4 mm (medullary) into the kidney to measure tissue PO_2_ using the Oxylite Pro (Oxford Optronix, United Kingdom). In a sub-set of CIH and sham animals (*n* = 7 per group), a laser speckle contrast imager (Moor FLPI-2, Moor Instruments, Devon United Kingdom) was positioned with the whole kidney in the field of view for assessment of renal microcirculatory perfusion (RP) (in addition to RBF, CPO_2_, and MPO_2_). After a 60-min equilibration period, a 5-min baseline period of RBF, arterial pressure, RP, and cortical and medullary PO_2_ was recorded for analysis. After measurement of baseline values, a series of ten 30 s episodes of intermittent asphyxia (IA) were initiated. Each IA episode was characterized by an F_i_O_2_ of 0.10 and F_i_CO_2_ 0.03 with the remaining balance made up of nitrogen. Each IA episode was followed by a 60 s recovery period breathing medical grade air (F_i_O_2_ 0.21, balance nitrogen). All variables were measured continuously during the IA protocol.

### Method of euthanasia

At the conclusion of the experimental protocol all rats were humanely euthanized. In accordance with standards set forth by the American Veterinary Medical Association and the Institutional Animal Care and Use Committee at Des Moines University Medicine and Health Sciences, all rats were euthanized via anesthetic overdose (5% isoflurane) combined with the addition of a secondary physical method of euthanasia.

### Measurement of renal mRNA expression

At the conclusion of physiological experiments and after anesthetic overdose, rat kidneys were excised and dissected, flash frozen in liquid nitrogen, and stored at −80°C for later analysis. Studies of renal mRNA expression were performed as previously described (Kious et al., 2022). Briefly, RNA isolation and cDNA synthesis were performed according to manufacturer instructions using the Direct-zol RNA MiniPrep Plus (Zymo, Irvine, CA) and High-Capacity cDNA Reverse Transcriptase (Thermo Fisher Scientific, Waltham, MA) kits, respectively. Gene expression was assessed by Sso Advanced SYBR green chemistry (Bio-Rad, Hercules, CA) real-time PCR following reverse transcription of total RNA. Real-time PCR was performed on a CFX Connect Real Time PCR Detection System (Bio-Rad, Hercules, CA). β-Actin mRNA was quantified as an internal control for each sample and quantifications were performed using the ΔΔCt method and expressed as fold change. Forward and Reverse primer sequences are show in Table 1. The following genes were probed in our samples: Krüppel-like factor 2 (KLF2), nuclear factor erythroid 2-related factor 2 (NRF2), copper zinc superoxide dismutase (CuZn SOD), NAD(P) H oxidase catalytic subunit (gp91phox), Krüppel-like Factor 15 (KLF15), E Cadherin, connective tissue growth factor (CTGF), tissue inhibitor of metalloproteinase 1 (TIMP), vascular endothelial growth factor (VEGF), Galectin-3, snail family zinc finger 1 (snail), and collagen I.

**TABLE 1:** Primer sequences.

Primer	Forward	Reverse
β-Actin	GGA​GAT​TAC​TGC​CCT​GGC​TCC​TA	GAC​TCA​TCG​TAC​TCC​TGC​TTG​CTG
KLF2	ACT​TGC​AGC​TAC​ACC​AAC​TG	CTGTGACCCGTGTGCTTG
NRF2	AAG​GTT​TCC​CAT​CTC​CAT​CAC	GAA​TAA​AGT​TGC​CGC​TCA​GAA
CuZnSOD	CGG​CCA​ATG​ATG​GAA​TGC​TC	GCA​GAA​GGC​AAG​CGG​TGA​AC
gp91phox	TTC​ACC​TAC​AGC​ACG​CTT​GTG	GAT​GAC​TGT​CTT​GCC​CCA​AGT​T
KLF15	CAG​CTT​CTG​GTC​AAC​ATC​CA	GAA​GTT​CTG​CTG​CTG​GGT​TC
E Cadherin	AAA​GCA​GGA​AGA​AAA​CAC​CAC​TC	AAA​GGG​CAC​GCT​ATC​AAC​ATT​AG
CTGF	CCT​GGT​CCA​GAC​CAC​AGA​GT	TTT​TCC​TCC​AGG​TCA​GCT​TC
TIMP1	GAC​CAC​CTT​ATA​CCA​GCG​TT	GTC​ACT​CTC​CAG​TTT​GCA​AG
VEGF	ATC​ATG​CGG​ATC​AAA​CCT​CAC​C	GGT​CTG​CAT​TCA​CAT​CTG​CTA​TGC
Galectin-3	GCA​CTA​ACC​AGG​AAA​ATG​GCA​GAC​G	CGC​TCA​TAA​CAC​ACA​GGG​CAG​TTC
snai1	AGC​CCA​ACT​ATA​GCG​AGC​TG	CCA​GGA​GAG​AGT​CCC​AGA​TG
collagen I	ATC​TCC​TGG​TGC​TGA​TGG​AC	ACC​TTG​TTT​GCC​AGG​TTC​AC

### Data analysis

**FIGURE 2:**
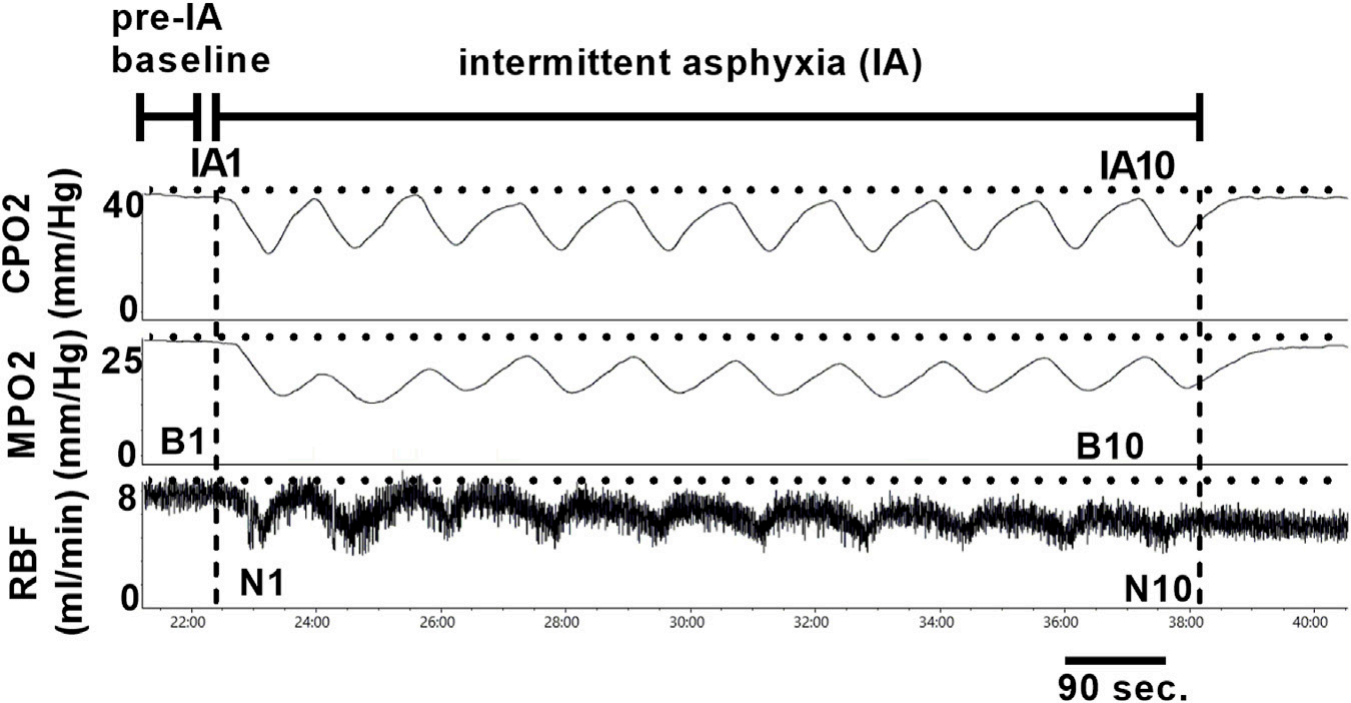
Glomerular filtration rate in sham and CIH rats after CIH conditioning. Shown in panel **(A)** are representative decay curves of transcutaneous fluorescence of FITC-Sinistrin in one CIH and one sham animal. Panel **(B)** illustrates mean data for GFR at the post-exposure time point. GFR was significantly increased in CIH relative to sham. Data analyzed by unpaired *t*-test and displayed as violin plots with median and quartiles, *n* = 10 animals per group. *p < 0.05 vs. sham.

All physiological parameters except GFR (MPD Lab, Medibeacon, St Louis, MO) and RP (moorFLPI2 Research Software, Moor Instruments, Devon, United Kingdom) were recorded with a Powerlab data acquisition and analysis system (AD Instruments, Colorado Springs, CO). Mean values for baseline (B1) RBF, RP, and CPO_2_ and MPO_2_ were calculated from 5-min of data immediately following a 60-min post-instrumentation equilibration period (pre-IA baseline). In addition to computing baseline values, we quantified the RBF, RP, and CPO_2_/MPO_2_ response to IA using the following approaches. Baseline (b2-b10) and nadir (n1-n10) measurements occurring during IA were based on 10 s periods centered around appropriate peaks and nadirs. First, we analyzed the change (if any) in normoxic baseline RBF, RP, CPO_2_, and MPO_2_ relative to the pre-IA baseline (e.g. B1-B2, B1-B3, B1-B4, etc.). Then we analyzed the average change in RBF, RP, and CPO_2_/MPO_2_ during the IA protocol (e.g. B1-N1, B2-N2, B3-N3, etc.). A graphical representation of the time points analyzed is shown in Figure 2. Group mean data was analyzed using unpaired t-tests. All data was analyzed using GraphPad Prism version 9.5.1 for Windows software (San Diego, CA), and the threshold for statistical significance was set at *p* < 0.05. Values in the results section are expressed as mean ± standard deviation, and in the figures as median and quartiles with violin plots.

## Results

### Effects of CIH on baseline hemodynamic variables, GFR, and renal tissue PO_2_

**FIGURE 3:**
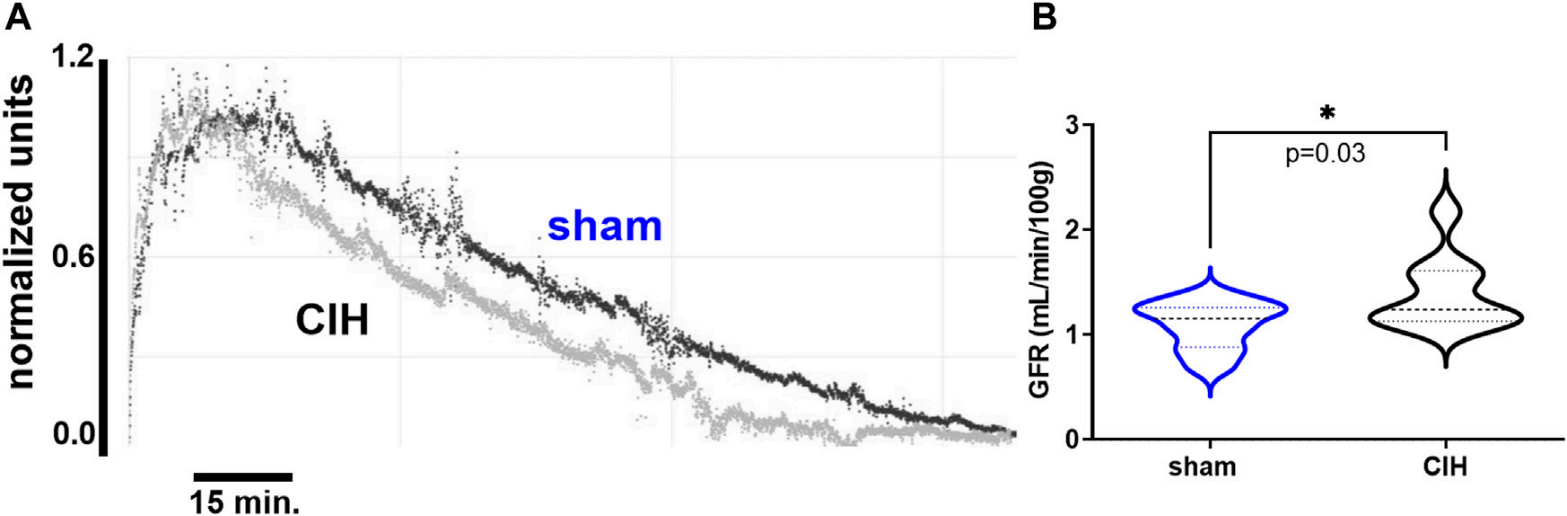
Representative tracings of renal tissue PO_2_ and renal artery blood flow during ten episodes of intermittent asphyxia. Time points for analysis are indicated in the figure and are as follows: 1. Pre-IA baseline (i.e., B1) 2. IA F_i_O_2_ 0.21 baseline deltas (e.g. B2 vs B1, B3 vs B1, *etc.*) 3. IA F_i_O_2_ 0.10/F_i_CO_2_ 0.03 deltas (e.g. B1 vs N1, B2 vs N2, B3 vs N3, *etc.*). IA = intermittent asphyxia, B = baseline, N = nadir.

As shown in Figure 3, after 2 weeks of CIH or sham conditioning, glomerular filtration rate (GFR) in conscious unrestrained rats was significantly higher in the CIH group than the sham group (1.09 ± 0.23 ml/min/g BW sham vs. 1.39 ± 0.34 ml/min/g BW CIH, t 2.29 df 18, *n* = 10 sham and CIH, *p* = 0.03). Baseline hemodynamic, CPO_2_, and MPO_2_ values are shown in Table 2. RBF (8.2 ± 3.3 ml/min sham vs. 4.6 ± 2.1 ml/min CIH, t 3.45 df 27, *n* = 15 sham, *n* = 14 CIH, *p* = 0.002), RVC (0.12 ± 0.03 ml/min/mmHg sham vs. 0.07 ± 0.05 ml/min/mmHg CIH, t 2.81 df 23, *n* = 13 sham, *n* = 12 CIH, *p* = 0.005), CPO_2_ (41.5 ± 8.4 mmHg sham vs. 31.0 ± 8.2 mmHg CIH, t 3.14 df 23, *n* = 14 sham, *n* = 11 CIH, *p* = 0.005), and MPO_2_ (29.8 ± 7.5 mmHg sham vs. 18.1 ± 2.5 mmHg CIH, t 5.71 df 25, *n* = 12 sham, *n* = 15 CIH, *p* = 0.005) were all lower in CIH vs sham. In contrast, arterial pressure was significantly higher in CIH vs sham animals (73.2 ± 14.3 mmHg sham vs. 87.1 ± 14.9 mmHg CIH, t 2.45 df 25, *n* = 15 sham, *n* = 12 CIH, *p* = 0.021). Heart rate was not significantly different between groups at baseline (315.3.2 ± 5.9 mmHg sham vs. 307.0 ± 35.6 mmHg CIH, t 0.74 df 25, *n* = 15 sham, *n* = 12 CIH, *p* = 0.021).

**TABLE 2:** Baseline measurements.

	Sham	CIH	*p*-Value
Renal Blood Flow (ml/min)	8.2 ± 3.3	4.6 ± 2.1*	*p* = 0.002
Mean Arterial Pressure (mmHg)	70.3 ± 15.6	87.1 ± 14.9*	*p* = 0.013
Heart Rate (beats/min)	315 ± 23	307 ± 36	*p* = 0.466
Renal Vascular Conductance (ml/min/mmHg)	0.12 ± 0.03	0.07 ± 0.05*	*p* = 0.005
Renal Cortical PO_2_ (mmHg)	41.5 ± 8.4	31.0 ± 8.2*	*p* = 0.005
Renal Medullary PO_2_ (mmHg)	29.8 ± 7.5	18.1 ± 2.5*	*p* < 0.0001

All data in this table was collected under anesthesia. Data was analyzed using unpaired t-tests. Sham and CIH, values are expressed as mean ± SD, *n* = 12–14 per animals group. *p < 0.05 vs. sham.

### Effects of IA on hemodynamic variables and tissue PO_2_ in CIH-conditioned rats

**FIGURE 4:**
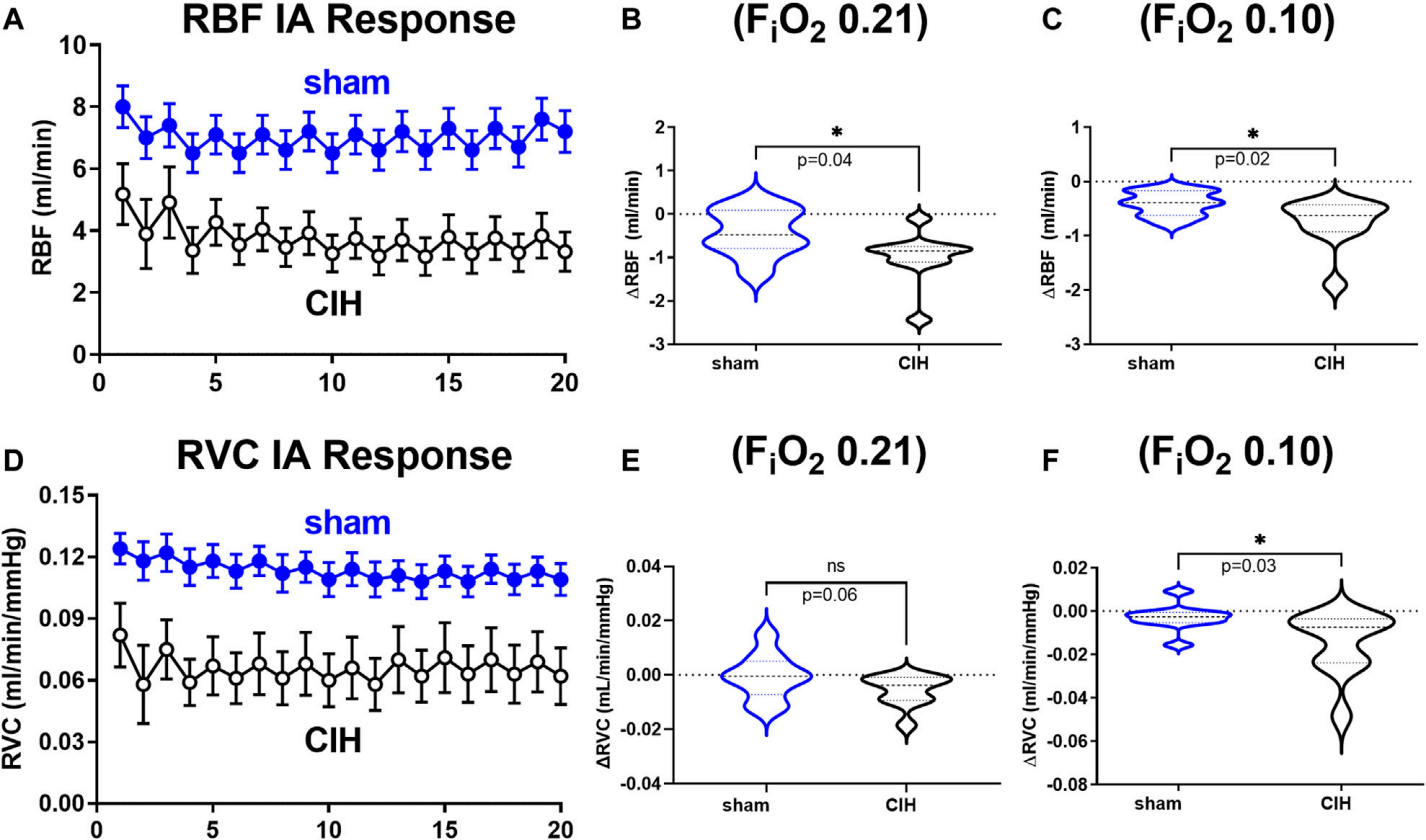
Renal artery blood flow and renal vascular conductance during intermittent asphyxia. Shown in panel **(A)** and **(D)** are composite traces of the renal artery blood flow (RBF) and renal vascular conductance (RVC) responses to ten episodes of intermittent asphyxia (IA, F_i_O_2_ 0.10, F_i_CO_2_ 0.03) in sham and CIH groups. The panels to the right show mean data for the 1) change in RBF **(B)** and RVC **(E)** during normoxia (F_i_O_2_ 0.21) relative to pre-IA baseline and 2) the change in RBF **(C)** and RVC **(F)** during asphyxia (F_i_O_2_ 0.10, F_i_CO_2_ 0.03). Data analyzed by unpaired *t*-test and displayed as violin plots with median and quartiles, *n* = 10 animals per group. *p < 0.05 vs. sham.

**FIGURE 5:**
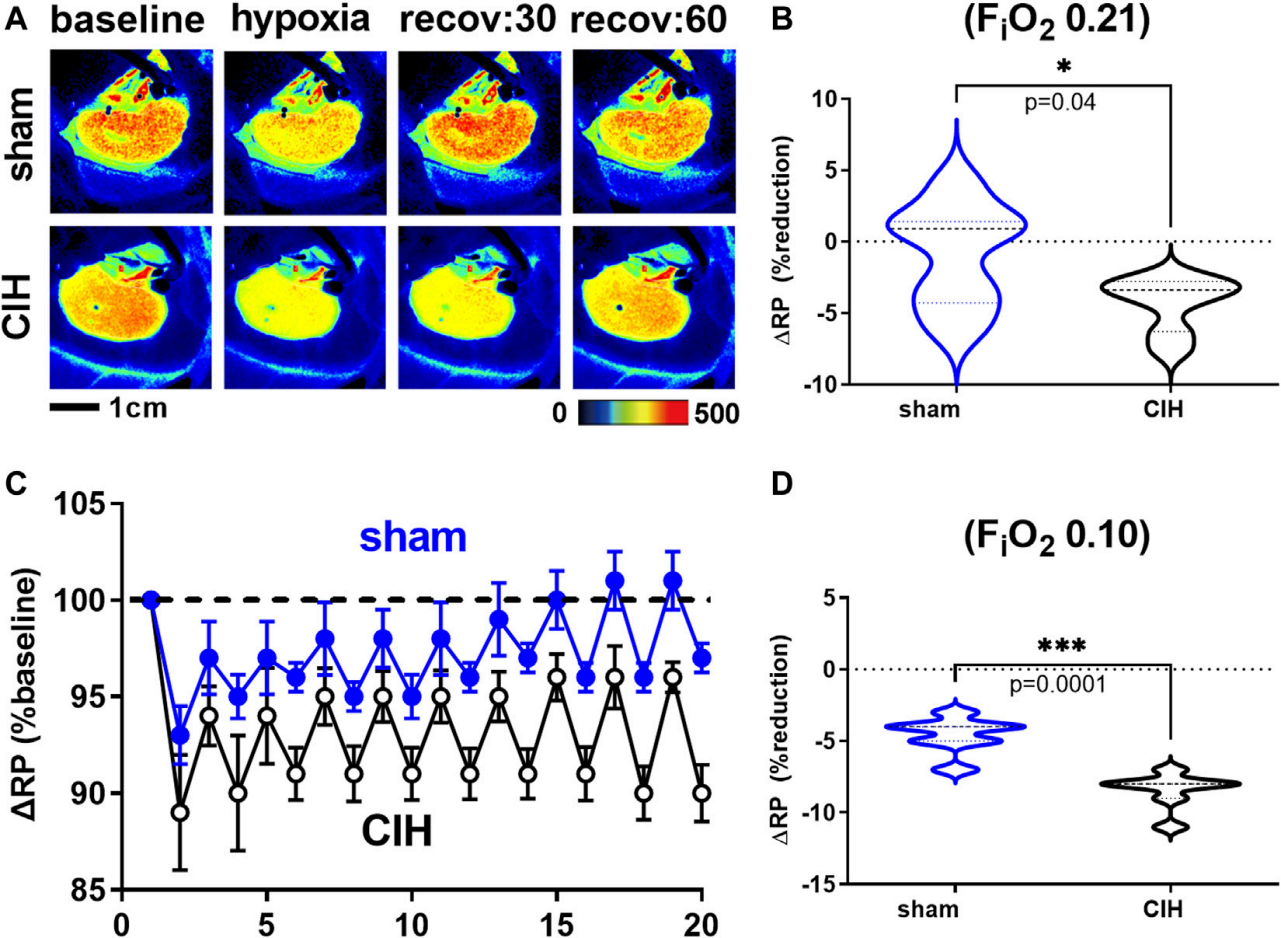
Renal microcirculatory perfusion during intermittent asphyxia. Shown in panel **(A)** are representative images of renal microcirculatory perfusion (RP) measured with laser speckle contrast imaging during exposure to a single episode of intermittent asphyxia (IA, F_i_O_2_ 0.10, F_i_CO_2_ 0.03) in one sham and one CIH animal. Shown in panel **(C)** are composite traces of the RP response to ten episodes of IA in sham and CIH groups. Panel **(B)** and **(D)** show mean data for the 1) change in RP during normoxia (F_i_O_2_ 0.21) relative to pre-IA baseline and 2) the change in RP during asphyxia (F_i_O_2_ 0.10, F_i_O_2_ 0.03), respectively. Data analyzed by unpaired *t*-test and displayed as violin plots with median and quartiles, *n* = 7 per group. ***p < 0.001, ****p < 0.0001 vs. sham.

**FIGURE 6:**
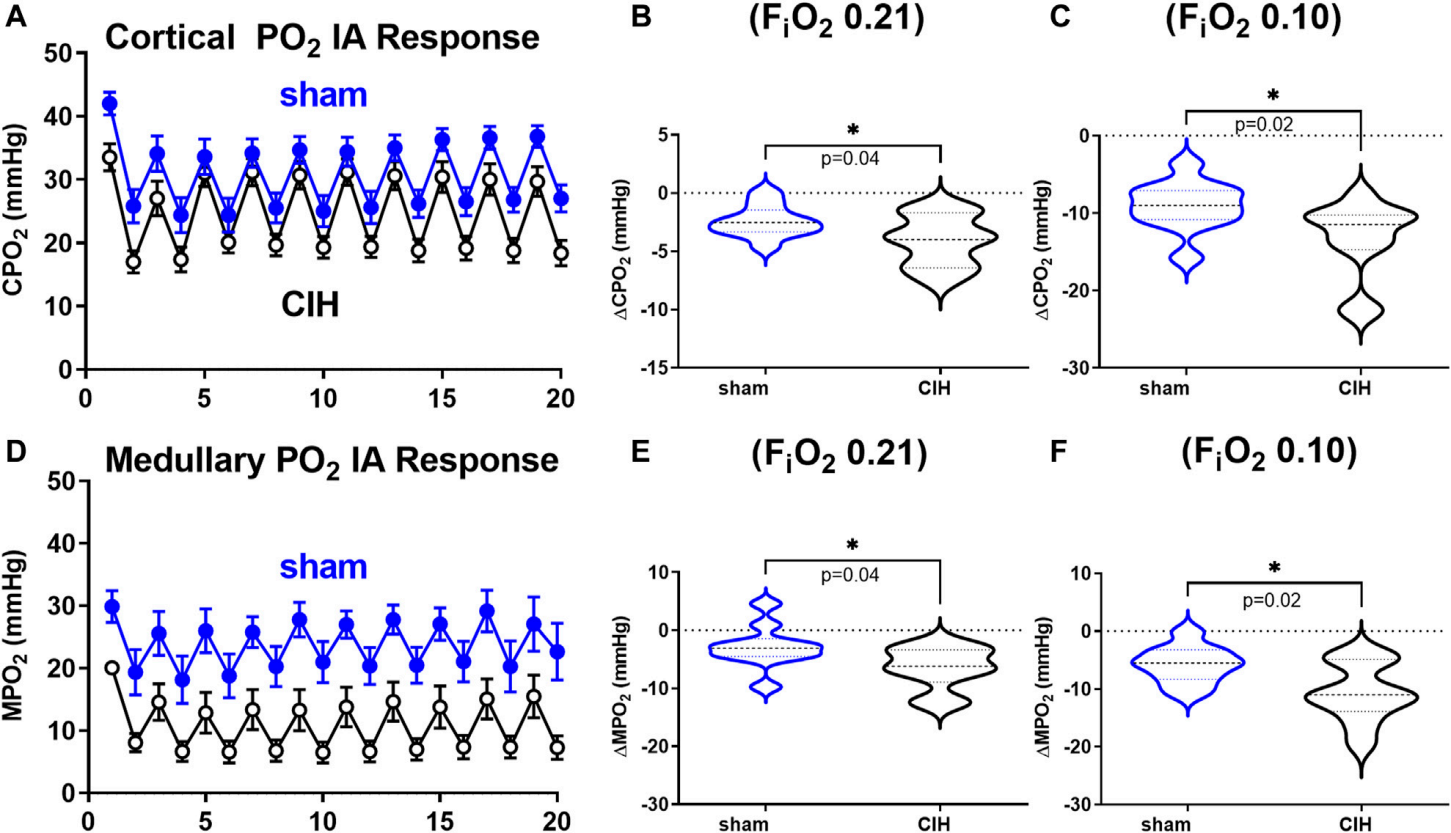
Cortical and medullary PO_2_ during intermittent asphyxia. Shown in panel **(A)** and **(D)** are composite traces of the cortical and medullary PO_2_ response to ten episodes of intermittent asphyxia (IA, F_i_O_2_ 0.10, F_i_CO_2_ 0.03) in sham and CIH groups. The panels to the right show mean data for the 1) change in cortical **(B)** and medullary **(E)** tissue PO_2_ during normoxia (F_i_O_2_ 0.21) relative to pre-IA baseline and 2) the change in cortical **(C)** and medullary **(F)** PO_2_ during IA exposures. Data analyzed by unpaired *t*-test and displayed as violin plots with median and quartiles, *n* = 10–11 per group. **p < 0.01, ***p < 0.001, ****p < 0.0001 vs. sham.

Hemodynamic and tissue PO_2_ responses to the IA protocol are shown in Figures 4–6. Normoxic RBF (-0.43 ± 0.54 ml/min sham vs. -0.98 ± 0.59 ml/min CIH, t 2.2 df 18, *n* = 10 per group, *p* = 0.04), RVC (-0.0006 ± 0.008 ml/min/mmHg sham vs. -0.006 ± 0.006 ml/min/mmHg CIH, t 1.6 df 18, *n* = 10 per group, *p* = 0.06), RP (-0.84% ± 3.6% sham vs. -4.33 ± 1.9% CIH, t 2.26 df 12, *n* = 7 per group, *p* = 0.04) CPO_2_ (-2.4 ± 1.4 mmHg sham vs. -4.1 ± 2.2 mmHg CIH, t 2.2 df 20, *n* = 11 per group, *p* = 0.04) and MPO_2_ (-2.8 ± 3.7 mmHg sham vs. -6.5 ± 3.6 mmHg CIH, t 2.2 df 18, *n* = 10 per group, *p* = 0.04) baselines during IA decreased relative to pre-IA baseline in both groups, but to a greater extent in CIH. Similarly, RBF (-0.41 ± 0.22 ml/min sham vs. -0.75 ± 0.45 ml/min CIH, t 2.2 df 13, *n* = 10 per group, *p* = 0.04), RVC (-0.002890 ± 0.006101 ml/min/mmHg sham vs. -0.01488 ± 0.01473 ml/min/mmHg CIH, t 2.4 df 18, *n* = 10 per group, *p* = 0.03), RP (-4.6 ± 1.3% sham vs. -8.4 ± 1.3% CIH, t 5.7 df12, *n* = 7 per group, *p* = 0.0001) and CPO_2_ (-9.0 ± 3.2 mmHg sham vs. -13.3 ± 5.0 mmHg CIH, t 2.4 df 20, *n* = 11 per group, *p* = 0.02) MPO_2_ (-5.8 ± 3.2 mmHg sham vs. -10.7 ± 5.0 mmHg CIH, t 2.6 df 18, *n* = 10 per group, *p* < 0.02) decreased during asphyxia in both groups, but to a greater extent in CIH vs sham.

### Effect of CIH on renal cortical mRNA expression

**FIGURE 7:**
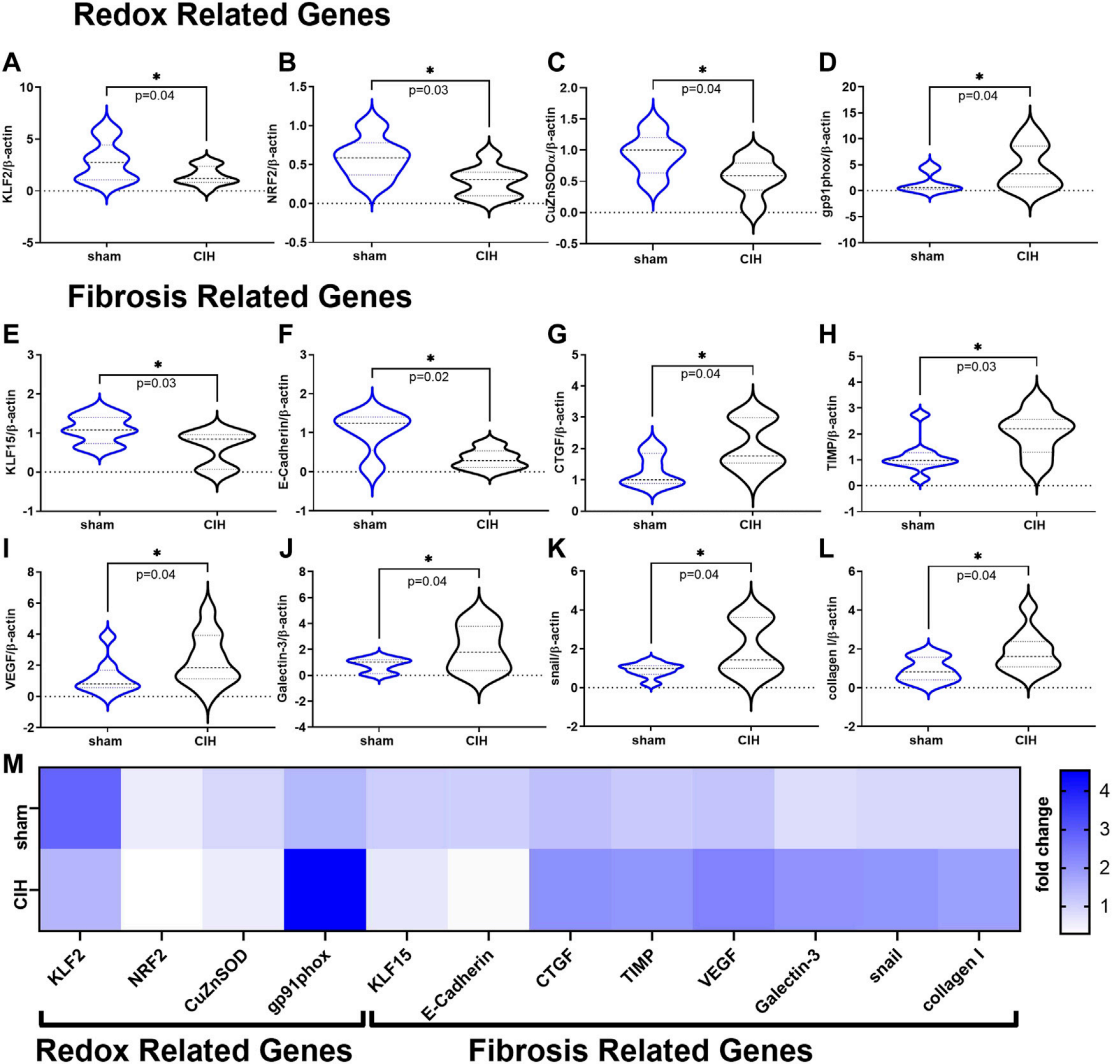
Redox and Fibrosis related mRNA expression in renal cortical tissue. Panels **(A–D)** show mean mRNA expression for redox genes **(A)** Krüppel-like factor 2 (KLF2), **(B)** nuclear erythroid 2-related factor 2 (NRF2), **(C)** copper zinc super oxide dismutase (CuZnSOD), and **(D)** the NADPH oxidase catalytic subunit (gp91phox). Panels **(E–L)** show mean mRNA expression for fibrosis related genes **(E)** Krüppel-like factor 15 (KLF15), **(F)** E-Cadherin **(G)** connective tissue growth factor (CTGF), **(H)** tissue inhibitor of metalloproteinase 1 (TIMP), **(I)** vascular endothelial growth factor (VEGF), **(J)** Galectin-3 **(K)** snail family zinc finger 1 (snail), and **(L)** collagen **I**. Panel M is a heat map showing differences in mean mRNA expression. All data is expressed as fold change relative to housekeeping gene β-actin. Data analyzed by unpaired *t*-test and displayed as violin plots with median and quartiles, *n* = 6–10 samples per group. *p < 0.05 vs. sham.

Results of qRT-PCR studies on renal cortical tissue are shown in Figure 7. Expression of genes related to antioxidant defense (KLF2 (2.81 ± 1.9-fold change sham vs. 1.46 ± 0.89-fold change CIH, t 2.2 df 19, *n* = 9–12 per group, *p* = 0.04) NRF2 (0.59 ± 0.26-fold change sham vs. 0.28 ± 0.19-fold change CIH, t 2.5 df 12, *n* = 6–8 per group, *p* = 0.03), CuZnSOD (0.93 ± 0.33-fold change sham vs. 0.55 ± 0.30-fold change CIH, t 2.2 df 13, *n* = 5–10 per group, *p* = 0.04) were downregulated while genes associated with the pro-oxidative catalytic subunit of NADPH oxidase (gp91phox (1.44 ± 1.8-fold change sham vs. 4.54 ± 4.2-fold change CIH, t 1.9 df 14, *n* = 8 per group, *p* = 0.04) were upregulated in CIH vs. sham. Expression of anti-fibrotic genes KLF15 (1.10 ± 0.35-fold change sham vs. 0.62 ± 0.44-fold change CIH, t 2.4 df 15, *n* = 8–9 per group, *p* = 0.02), and E-Cadherin (1.03 ± 0.54-fold change sham vs. 0.32 ± 0.25-fold change CIH, t 2.8 df 9, *n* = 5–6 per group, *p* = 0.02) were lower in CIH vs sham, whereas expression of pro-fibrotic/fibrosis-associated genes (CTGF (1.29 ± 0.51-fold change sham vs. 2.10 ± 0.79-fold change CIH, t 2.3 df 13, *n* = 7–8 per group, *p* = 0.04), TIMP (1.13 ± 0.22-fold change sham vs. 2.04 ± 0.83-fold change CIH, t 2.4 df 14, *n* = 7–9 per group, *p* = 0.03), VEGF (1.22 ± 1.13-fold change sham vs. 2.35 ± 1.62-fold change CIH, t 1.8 df 18, *n* = 9–11 per group, *p* = 0.04), Galectin 3 (0.81 ± 0.54-fold change sham vs. 2.08 ± 1.74-fold change CIH, t 1.9 df 12, *n* = 7 per group, *p* = 0.04), snail (0.91 ± 0.37-fold change sham vs. 2.02 ± 1.38-fold change CIH, t 2.2 df 14, *n* = 8 per group, *p* = 0.04), and collagen I (0.91 ± 0.60-fold change sham vs. 1.86 ± 1.05-fold change CIH, t 2.2 df 15, *n* = 7–10 per group, *p* = 0.04) were higher in CIH vs. sham.

## Discussion

In this study we sought to determine the effect of CIH conditioning on renal blood flow (RBF), renal vascular conductance (RVC), renal microcirculatory perfusion (RP), glomerular filtration rate (GFR), and renal cortical and medullary tissue PO_2_ (CPO_2_, MPO_2_) at rest and during exposure to intermittent asphyxia (IA). The main findings of our study are (I) GFR measured in the conscious state was significantly increased in CIH rats vs sham rats, (II) baseline RBF, RVC, CPO_2_, and MPO_2_ were significantly reduced in CIH rats vs sham rats, (III) normoxic RBF, RVC, RP, CPO_2_, and MPO_2_ decreased below baseline during the course of IA exposure, (IV) RBF, RVC, CPO_2_, and MPO_2_ decreased to a greater extent in CIH rats vs sham rats during the hypoxic/hypercapnic (asphyxia) portion of the IA protocol, (V) CIH was associated with activation of pro-oxidative and pro-fibrotic gene programs. Our results indicate that the CIH has significant effects on control of renal hemodynamics and tissue oxygenation during normoxia and exposure to asphyxia.

### Effects of chronic intermittent hypoxia on GFR

We observed that GFR was higher in CIH relative to sham, an observation consistent with the hyperfiltration observed in patient populations with SA (Kinebuchi et al., 2004; Adeseun and Rosas, 2010). In contrast, O’Neil et al. found a lower GFR in CIH conditioned rats relative to sham (O'Neill et al., 2019), an observation which is also consistent with clinical studies showing reduced GFR in patients with SA (Li et al., 2019). Given the similarities of the intensity and duration of the CIH paradigms used in our study and that of O’Neil et al. it is difficult to reconcile these differences. It may be that our findings represent a hyperfiltration state which precedes eventual decreases in GFR associated with prolonged or more severe CIH exposure. Progression from hyperfiltration to reduced GFR occurs in diabetic nephropathy (Palatini, 2012), so this phenomenon is not without precedent.

Another explanation may be the physiological state under which GFR was measured in these two studies. In our study, GFR was measured in conscious unrestrained animals, whereas the study by O’Neil et al. measured GFR in pentobarbital anesthetized rats (O'Neill et al., 2019). Previous studies have shown that pentobarbital anesthesia in rats causes a marked reduction in mean arterial pressure, heart rate, GFR, and effective renal plasma flow (Walker et al., 1986). Determining whether or not the differences in the effect of CIH on GFR observed between studies can be attributed to anesthesia will require future studies to perform both techniques in the same animals. While our studies do not directly address mechanisms of glomerular hyperfiltration after CIH conditioning there are multiple physiological changes occurring with CIH that may contribute. Vascular and autonomic dysfunction associated with CIH are mediated in part by chronic RAAS activation (Marcus et al., 2010; Marcus et al., 2012), which has been shown to elicit constriction of the efferent arterioles and increase GFR (Cortinovis et al., 2022). Further study is needed to fully address the nature and time course of changes in GFR with CIH conditioning.

### Effects of chronic intermittent hypoxia on RBF, RVC, and RP

The present study shows significantly lower baseline RBF, RVC, and RP as well as exaggerated hypoxia-evoked reductions in RBF, RVC, and RP in CIH relative to sham. The only study that has previously explored the effect of CIH conditioning on baseline RBF found no significant difference in RBF or renal vascular resistance (RVR) between CIH and sham groups (O'Neill et al., 2019). This unexpected finding may be explained by the nature of CIH related changes in control of organ blood flow. CIH contributes to enhanced baseline sympathetic nerve activity and increased arterial pressure (Huang et al., 2009; Gilmartin et al., 2010; Marcus et al., 2010; Del Rio et al., 2016), as well as enhanced sympathetic responses to hypoxic events (Huang et al., 2009; Gilmartin et al., 2010; Marcus et al., 2010). CIH specifically augments chemoreflex control of renal sympathetic nerve activity (Huang et al., 2009) (RSNA), and previous work has established that conditions characterized by chemoreflex-mediated increases in RSNA have the potential to cause reductions in RBF and RVC, and increases in RVR (Clayton et al., 2011; Marcus et al., 2015; Pügge et al., 2016; Kious et al., 2022). Thus in a CIH model it would be logical to hypothesize reductions in RBF and RVC. Both our study and the study by O’Neil et al. measured RBF under anesthesia, however as mentioned previously, O’Neil et al. used pentobarbital anesthesia which has been shown to obtund cardiovascular reflex function (Peiss and Manning, 1964). Therefore, it is possible that pentobarbital suppressed chemoreflex control of RSNA and for this reason the expected reductions in RBF after CIH were not observed by O’Neil et al. In addition to changes in autonomic function that affect control of blood flow, vascular dysfunction also likely plays a role in the dysregulation of integrated hemodynamic control we observed. We have previously shown that CIH is associated with vascular endothelial dysfunction (Philippi et al., 2010; Marcus et al., 2012) and oxidative stress (Marcus et al., 2010; Philippi et al., 2010), however these parameters have not previously been assessed in the renal vasculature in the CIH model. ROS may be generated in CIH animals as a results of mitochondrial dysfunction (Prabhakar et al., 2007), or acutely in response to hypoxia, angiotensin II (ANGII), and/or proinflammatory cytokines (Liu et al., 2017). CIH is associated with RAAS activation, renal inflammation, and oxidative damage in the kidney (Sun et al., 2012). ROS in the kidney has previously been shown to reduce renal medullary blood flow and to induce afferent arteriolar constriction in presence of ANGII (Ratliff et al., 2016). Thus, the multiple pathophysiological insults associated with CIH are consistent with established causes of autonomic and vascular dysfunction, and the observed dysregulation of renal hemodynamic control observed in our studies.

### Effects of chronic intermittent hypoxia on renal tissue PO_2_

Previous studies have shown a reduction in resting normoxic renal cortical but not medullary tissue PO_2_ after conditioning with a similar CIH paradigm (O’Neill et al., 2019). Interestingly, this occurred in the presence of a reduction in GFR and sodium transport without a reduction in renal artery blood flow. In addition, the authors noted that CIH animals exhibited higher renal oxygen consumption at any given level of sodium transport. Thus, in this study decreases in cortical tissue PO_2_ were driven by changes in tissue metabolism rather than as a result of decreased oxygen delivery. In the current study, both cortical and medullary PO_2_ were lower in CIH exposed animals at baseline and during the IA protocol. While the reason for this discrepancy is unclear, one potential explanation might center around differences in GFR between the studies. In our study we observed increased GFR and decreased RBF in CIH animals under normoxic conditions, whereas O’Neil et al. observed decreased GFR and sodium transport in CIH animals relative to sham. While O’Neil et al. observed no differences in oxygen consumption between CIH and sham, this was in the face of lower GFR and sodium transport. It is possible that a higher GFR in our study drove greater sodium transport and oxygen consumption contributing to lower tissue PO_2_. We did not measure tissue oxygen consumption or sodium transport, so our data can neither directly support nor refute this possibility, but it is a plausible explanation based on the data we collected.

Under normal circumstances, renal tissue PO_2_ is maintained at stable levels by a unique interaction between RBF, GFR, and oxygen consumption (QO_2_) (Brezis et al., 1994). Renal QO_2_ is primarily driven by the metabolic demands associated with tubular solute reabsorption, however this relationship can become dysregulated as a result of mitochondrial dysfunction, RAAS activation, and hypoxic conditions (Brezis et al., 1994; Haase, 2013). Disease states such as diabetes and hypertension are often associated with increased QO_2_ and decreased renal tissue PO_2_ (Hansell et al., 2013). In these diseases, elevated kidney QO_2_ is commonly mediated by increased angiotensin receptor tonus and oxidative stress which contributes to progression of renal damage and reduced function (Hansell et al., 2013). Given the well-known RAAS activation and oxidative stress in CIH models of SA, these factors likely contribute to the decreases we observed in renal tissue PO_2_. Thus, the combination of oscillations in RSNA and RBF/RP combined with persistent RAAS activation and intermittent hypoxemia (associated with individual episodes of asphyxia) act synergistically to create a state of tissue hypoxia.

### Effect of CIH on renal gene expression

Because we observed notable decreases in RBF/RVC/RP in CIH conditioned animals, we sought to determine how this might affect gene expression of transcription factors related to blood flow. We measured expression of the shear stress-sensitive transcription factor Krüppel-like factor 2 (KLF2), as KLF2 controls expression of mediators of antioxidant defense, nitric oxide production, and tissue fibrosis (Dekker et al., 2005; Boon et al., 2007; Fledderus et al., 2008) and has previously been shown to be downregulated in animal models characterized by reduced RBF (Haack et al., 2014; Del Rio et al., 2017; Marcus et al., 2018; Kious et al., 2022). As shown in Figure 7, we observed that the gene expression of renal cortical KLF2 and the master antioxidant transcription factor NRF2 (a downstream target of KLF2) were significantly downregulated in CIH relative to sham. To determine if NRF2 downregulation resulted in decreased gene expression of antioxidant enzymes under its control, we measured mRNA expression of CuZn SOD, which was also significantly decreased in CIH vs sham. In contrast, gene expression of the catalytic subunit of NADPH oxidase (gp91phox) was significantly increased in CIH vs sham, consistent with findings in other studies (Prabhakar et al., 2007; Sun et al., 2012).

Tissue fibrosis has been theorized to be a common pathway to renal dysfunction (Wang et al., 2022) and may resist oxygen diffusion and contribute to tissue hypoxia (Edwards and Kurtcuoglu, 2022), so we examined gene expression of fibrosis related pathways in renal cortical tissue. In particular we focused on pathways related to tissue hypoxia because of its relevance to our physiological measurements. KLF15, and E-Cadherin, which have fibrosis-suppressing activity and have been shown to be downregulated by ANG II and hypoxia (Esteban et al., 2006; Lu et al., 2019), were decreased in cortical tissue from CIH relative to sham. Expression of pro-fibrotic CTGF, TIMP, Galectin 3, Snail, and VEGF (all of which are induced by hypoxia and/or ANG II (Liu et al., 2017; Boutin et al., 2022; Khalaji et al., 2023)), was significantly higher in cortical tissue from CIH vs sham, suggesting that CIH exposure promotes activation of pro-oxidative, pro-fibrotic signaling that may contribute to tissue damage and organ dysfunction.

### Strengths and limitations

Often, discerning the effects of multiple-concurrent co-morbidities or conditions can make clear interpretation of data difficult. The common presence of obesity, metabolic syndrome, and advanced age alongside sleep apnea, can confound interpretation of the relative contribution of any given variable. Our animal model examines the physiological changes that occur in response to CIH conditioning in the absence of common co-morbidities, giving a clearer picture of the contribution of CIH *per se*. These findings yield significant insight into the potential for the CIH associated with sleep apnea to precipitate or hasten the decline of renal function and to identify the mechanisms by which this might occur. In addition to the specificity of our model, our approach also has significant advantages. In this study we combine measurement of renal artery blood flow using a transit time flow probe with measurement of renal microcirculatory perfusion using laser speckle contrast imaging (LSCI). The larger imaging field used in LSCI allows for a more comprehensive quantification of ‘whole organ’ regulation of microcirculatory perfusion.

In this study direct measurement of RBF, RVC, RP, CPO_2_, and MPO_2_ is made at the conclusion of the experimental period under anesthesia. The use of anesthesia during invasive experiments is a limitation, however, any changes induced by anesthesia would be expected to affect both experimental and control animals, thus we do not feel this confounds interpretation of differences observed between groups. Measurement of physiological variables under anesthesia has inherent limitations regardless of anesthetic, but we feel that the difference in anesthetics used in similar studies (O’Neill et al., 2019) should be considered when comparing data. In addition, we acknowledge that the length of our CIH exposure (2 weeks) is short relative to the amount of time that patients with sleep apnea are typically exposed to CIH associated with their condition. Previous studies in rats and mice have shown that short-term CIH conditioning is sufficient to elicit enhanced chemoreflex control of sympathetic nerve activity (Huang et al., 2009; Marcus et al., 2010), however longer-term studies might reveal more profound effects of CIH on renal perfusion, tissue PO_2_, and injury. Indeed, studies focused on dose response of CIH exposure time show marked changes in tissue redox regulation and fibrosis with longer exposure times (Sun et al., 2012).

Finally, in the studies presented we only assessed GFR, renal hemodynamic regulation, and tissue PO_2_ in male animals, therefore inferences based on our study should be limited to the male sex. It is unclear how female sex steroid hormones interact with the insults associated with CIH, and therefore the effect of CIH on renal function and hemodynamic control in females deserves study.

## Contribution to the field

Renal dysfunction is a prominent independent risk factor for cardiovascular mortality (Centers for Disease Control and Prevention, 2021), and despite well-established epidemiological relationships between SA and renal dysfunction (Owada et al., 2013; Puckrin et al., 2015; Huang et al., 2017; Li et al., 2019), there is a paucity of mechanistic physiological studies in this area. A better understanding of the underlying physiological and biochemical changes that occur in the kidney in SA is important to address existing knowledge gaps. These studies provide new mechanistic insights into the pathophysiology of renal dysfunction in the context of SA. Because SA is a common co-morbidity in many cardio-metabolic diseases, our studies have the potential to promote greater understanding of the pathophysiology of renal dysfunction in clinical populations including type II diabetes, hypertension, and heart failure.

## Conclusion

Cumulatively, our results underscore the importance of CIH experienced during SA and its potential deleterious effects on regulation of renal function. These findings suggest that SA may contribute to renal damage and dysfunction as a result of promoting persistent renal hypoperfusion and tissue hypoxia both during wakefulness and during sleep when apneic episodes are occurring. Therapeutic modalities that reduce SA incidence and/or severity or that attenuate the deleterious effects of reductions in renal blood flow and chronic tissue hypoxia have the potential to improve renal health and function in patients with primary or co-morbid sleep apnea.

## Data Availability

The raw data supporting the conclusion of this article will be made available by the authors, without undue reservation.

## References

[B1] Adeseun G. A. Rosas S. E. (2010). The impact of obstructive sleep apnea on chronic kidney disease. Curr. Hypertens. Rep. 12 (5), 378–383. 10.1007/s11906-010-0135-1 20676805 PMC2975904

[B2] Boon R. A. Fledderus J. O. Volger O. L. van Wanrooij E. J. Pardali E. Weesie F. (2007). KLF2 suppresses TGF-beta signaling in endothelium through induction of Smad7 and inhibition of AP-1. Arterioscler. Thromb. Vasc. Biol. 27 (3), 532–539. 10.1161/01.ATV.0000256466.65450.ce 17194892

[B3] Boutin L. Dépret F. Gayat E. Legrand M. Chadjichristos C. E. (2022). Galectin-3 in kidney diseases: From an old protein to a new therapeutic target. Int. J. Mol. Sci. 23 (6), 3124. 10.3390/ijms23063124 35328545 PMC8952808

[B4] Brezis M. Agmon Y. Epstein F. H. (1994). Determinants of intrarenal oxygenation. I. Effects of diuretics. Am. J. Physiol. 267 (6), F1059–F1062. 10.1152/ajprenal.1994.267.6.F1059 7810692

[B5] Centers for Disease Control and Prevention (2021). Chronic kidney disease in the United States, 2021. Atlanta, GA: US Department of Health and Human Services, Centers for Disease Control and Prevention.

[B6] Clayton S. C. Haack K. K. Zucker I. H. (2011). Renal denervation modulates angiotensin receptor expression in the renal cortex of rabbits with chronic heart failure. Am. J. Physiol. Ren. Physiol. 300 (1), F31–F39. 10.1152/ajprenal.00088.2010 PMC3023215 20962112

[B7] Cortinovis M. Perico N. Ruggenenti P. Remuzzi A. Remuzzi G. (2022). Glomerular hyperfiltration. Nat. Rev. Nephrol. 18 (7), 435–451. 10.1038/s41581-022-00559-y 35365815

[B8] Dekker R. J. van Thienen J. V. Rohlena J. de Jager S. C. Elderkamp Y. W. Seppen J. (2005). Endothelial KLF2 links local arterial shear stress levels to the expression of vascular tone-regulating genes. Am. J. Pathol. 167 (2), 609–618. 10.1016/S0002-9440(10)63002-7 16049344 PMC1603569

[B9] Del Rio R. Andrade D. C. Lucero C. Arias P. Iturriaga R. (2016). Carotid body ablation abrogates hypertension and autonomic alterations induced by intermittent hypoxia in rats. Hypertension 68 (2), 436–445. 10.1161/HYPERTENSIONAHA.116.07255 27381902

[B10] Del Rio R. Andrade D. C. Toledo C. Diaz H. S. Lucero C. Arce-Alvarez A. (2017). Carotid body-mediated chemoreflex drive in the setting of low and high output heart failure. Sci. Rep. 7 (1), 8035. 10.1038/s41598-017-08142-3 28808320 PMC5556057

[B11] Di Murro A. Petramala L. Cotesta D. Zinnamosca L. Crescenzi E. Marinelli C. (2010). Renin-angiotensin-aldosterone system in patients with sleep apnoea: Prevalence of primary aldosteronism. J. Renin Angiotensin Aldosterone Syst. 11 (3), 165–172. 10.1177/1470320310366581 20488824

[B12] Edwards A. Kurtcuoglu V. (2022). Renal blood flow and oxygenation. Pflugers Arch. 474 (8), 759–770. 10.1007/s00424-022-02690-y 35438336 PMC9338895

[B13] Esteban M. A. Tran M. G. Harten S. K. Hill P. Castellanos M. C. Chandra A. (2006). Regulation of E-cadherin expression by VHL and hypoxia-inducible factor. Cancer Res. 66 (7), 3567–3575. 10.1158/0008-5472.CAN-05-2670 16585181

[B14] Fledderus J. O. Boon R. A. Volger O. L. Hurttila H. Ylä-Herttuala S. Pannekoek H. (2008). KLF2 primes the antioxidant transcription factor Nrf2 for activation in endothelial cells. Arterioscler. Thromb. Vasc. Biol. 28 (7), 1339–1346. 10.1161/ATVBAHA.108.165811 18467642

[B15] Friedemann J. Heinrich R. Shulhevich Y. Raedle M. William-Olsson L. Pill J. (2016). Improved kinetic model for the transcutaneous measurement of glomerular filtration rate in experimental animals. Kidney Int. 90 (6), 1377–1385. 10.1016/j.kint.2016.07.024 27665115

[B16] Gilmartin G. S. Lynch M. Tamisier R. Weiss J. W. (2010). Chronic intermittent hypoxia in humans during 28 nights results in blood pressure elevation and increased muscle sympathetic nerve activity. Am. J. Physiol. Heart Circ. Physiol. 299 (3), H925–H931. 10.1152/ajpheart.00253.2009 20581089 PMC4116417

[B17] Grundy D. (2015). Principles and standards for reporting animal experiments in the journal of Physiology and experimental Physiology. J. Physiol. 593 (12), 2547–2549. 10.1113/JP270818 26095019 PMC4500341

[B18] Haack K. K. Marcus N. J. Del Rio R. Zucker I. H. Schultz H. D. (2014). Simvastatin treatment attenuates increased respiratory variability and apnea/hypopnea index in rats with chronic heart failure. Hypertension 63 (5), 1041–1049. 10.1161/HYPERTENSIONAHA.113.02535 24516105 PMC3993007

[B19] Haase V. H. (2013). Mechanisms of hypoxia responses in renal tissue. J. Am. Soc. Nephrol. 24 (4), 537–541. 10.1681/ASN.2012080855 23334390

[B20] Hansell P. Welch W. J. Blantz R. C. Palm F. (2013). Determinants of kidney oxygen consumption and their relationship to tissue oxygen tension in diabetes and hypertension. Clin. Exp. Pharmacol. Physiol. 40 (2), 123–137. 10.1111/1440-1681.12034 23181475 PMC3951849

[B21] Herrera Pérez Z. Weinfurter S. Gretz N. (2016). Transcutaneous assessment of renal function in conscious rodents. J. Vis. Exp. (109), e53767. 10.3791/53767 27078159 PMC4841314

[B22] Hu Y. Mai L. Luo J. Shi W. Xiang H. Song S. (2022). Peripheral blood oxidative stress markers for obstructive sleep apnea-a meta-analysis. Sleep. Breath. 26 (4), 2045–2057. 10.1007/s11325-021-02557-z 34981298

[B23] Huang H. C. Walters G. Talaulikar G. Figurski D. Carroll A. Hurwitz M. (2017). Sleep apnea prevalence in chronic kidney disease - association with total body water and symptoms. BMC Nephrol. 18 (1), 125. 10.1186/s12882-017-0544-3 28376734 PMC5381077

[B24] Huang J. Lusina S. Xie T. Ji E. Xiang S. Liu Y. (2009). Sympathetic response to chemostimulation in conscious rats exposed to chronic intermittent hypoxia. Respir. Physiol. Neurobiol. 166 (2), 102–106. 10.1016/j.resp.2009.02.010 19429526

[B25] Khalaji A. Amirkhani N. Sharifkashani S. Behnoush A. H. (2023). Role of galectin-3 as a biomarker in obstructive sleep apnea: A systematic review and meta-analysis. Sleep. Breath. [Epub ahead of print] 10.1007/s11325-023-02842-z 37129844

[B26] Kinebuchi S. Kazama J. J. Satoh M. Sakai K. Nakayama H. Yoshizawa H. (2004). Short-term use of continuous positive airway pressure ameliorates glomerular hyperfiltration in patients with obstructive sleep apnoea syndrome. Clin. Sci. (Lond) 107 (3), 317–322. 10.1042/CS20040074 15191364

[B27] Kious K. W. Philipose A. Smith L. J. Kemble J. P. Twohey S. C. E. Savage K. (2022). Peripheral chemoreflex modulation of renal hemodynamics and renal tissue PO2 in chronic heart failure with reduced ejection fraction. Front. Physiol. 13, 955538. 10.3389/fphys.2022.955538 36091359 PMC9459040

[B28] Li X. Liu C. Zhang H. Zhang J. Zhao M. Sun D. (2019). Effect of 12-month nasal continuous positive airway pressure therapy for obstructive sleep apnea on progression of chronic kidney disease. Med. Baltim. 98 (8), e14545. 10.1097/MD.0000000000014545 PMC6407975 30813163

[B29] Lin C. H. Lurie R. C. Lyons O. D. (2020). Sleep apnea and chronic kidney disease: A state-of-the-art review. Chest 157 (3), 673–685. 10.1016/j.chest.2019.09.004 31542452

[B30] Liu M. Ning X. Li R. Yang Z. Yang X. Sun S. (2017). Signalling pathways involved in hypoxia-induced renal fibrosis. J. Cell. Mol. Med. 21 (7), 1248–1259. 10.1111/jcmm.13060 28097825 PMC5487923

[B31] Lu Y. Y. Li X. D. Zhou H. D. Shao S. He S. Hong M. N. (2019). Transactivation domain of Krüppel-like factor 15 negatively regulates angiotensin II-induced adventitial inflammation and fibrosis. FASEB J. 33 (5), 6254–6268. 10.1096/fj.201801809R 30776250

[B32] Maier L. E. Matenchuk B. A. Vucenovic A. Sivak A. Davenport M. H. Steinback C. D. (2022). Influence of obstructive sleep apnea severity on muscle sympathetic nerve activity and blood pressure: A systematic review and meta-analysis. Hypertension 79 (9), 2091–2104. 10.1161/HYPERTENSIONAHA.122.19288 35766054

[B33] Marcus N. J. Del Rio R. Ding Y. Schultz H. D. (2018). KLF2 mediates enhanced chemoreflex sensitivity, disordered breathing, and autonomic dysregulation in heart failure. J. Physiol. 596 (15), 3171–3185. 10.1113/JP273805 29023738 PMC6068211

[B34] Marcus N. J. Li Y. L. Bird C. E. Schultz H. D. Morgan B. J. (2010). Chronic intermittent hypoxia augments chemoreflex control of sympathetic activity: Role of the angiotensin II type 1 receptor. Respir. Physiol. Neurobiol. 171 (1), 36–45. 10.1016/j.resp.2010.02.003 20153844 PMC2846996

[B35] Marcus N. J. Olson E. B. Jr Bird C. E. Philippi N. R. Morgan B. J. (2009). Time-dependent adaptation in the hemodynamic response to hypoxia. Respir. Physiol. Neurobiol. 165 (1), 90–96. 10.1016/j.resp.2008.10.013 19013546 PMC2662762

[B36] Marcus N. J. Philippi N. R. Bird C. E. Li Y. L. Schultz H. D. Morgan B. J. (2012). Effect of AT1 receptor blockade on intermittent hypoxia-induced endothelial dysfunction. Respir. Physiol. Neurobiol. 183 (2), 67–74. 10.1016/j.resp.2012.05.025 22728949 PMC3409315

[B37] Marcus N. J. Pügge C. Mediratta J. Schiller A. M. Del Rio R. Zucker I. H. (2015). Exercise training attenuates chemoreflex-mediated reductions of renal blood flow in heart failure. Am. J. Physiol. Heart Circ. Physiol. 309 (2), H259–H266. 10.1152/ajpheart.00268.2015 26001414 PMC4504964

[B38] Molkov Y. I. Zoccal D. B. Moraes D. J. Paton J. F. Machado B. H. Rybak I. A. (2011). Intermittent hypoxia-induced sensitization of central chemoreceptors contributes to sympathetic nerve activity during late expiration in rats. J. Neurophysiol. 105 (6), 3080–3091. 10.1152/jn.00070.2011 21471394 PMC3118734

[B39] O'Neill J. Jasionek G. Drummond S. E. Brett O. Lucking E. F. Abdulla M. A. (2019). Renal cortical oxygen tension is decreased following exposure to long-term but not short-term intermittent hypoxia in the rat. Am. J. Physiol. Ren. Physiol. 316 (4), F635–F645. 10.1152/ajprenal.00254.2018 30648908

[B40] Orrù G. Storari M. Scano A. Piras V. Taibi R. Viscuso D. (2020). Obstructive Sleep Apnea, oxidative stress, inflammation, and endothelial dysfunction-An overview of predictive laboratory biomarkers. Eur. Rev. Med. Pharmacol. Sci. 24 (12), 6939–6948. 10.26355/eurrev_202006_21685 32633387

[B41] Ostrowski D. Heesch C. M. Kline D. D. Hasser E. M. (2023). Nucleus tractus solitarii is required for the development and maintenance of phrenic and sympathetic long-term facilitation after acute intermittent hypoxia. Front. Physiol. 14, 1120341. 10.3389/fphys.2023.1120341 36846346 PMC9949380

[B42] Owada T. Yoshihisa A. Yamauchi H. Iwaya S. Suzuki S. Yamaki T. (2013). Adaptive servoventilation improves cardiorenal function and prognosis in heart failure patients with chronic kidney disease and sleep-disordered breathing. J. Card. Fail 19 (4), 225–232. 10.1016/j.cardfail.2013.03.005 23582088

[B43] Palatini P. (2012). Glomerular hyperfiltration: A marker of early renal damage in pre-diabetes and pre-hypertension. Nephrol. Dial. Transpl. 27 (5), 1708–1714. Epub 2012 Mar 19. 10.1093/ndt/gfs037 22431709

[B44] Peiss C. N. Manning J. W. (1964). Effects of sodium pentobarbital on electrical and reflex activation of the cardiovascular system. Circ. Res. 14, 228–235. 10.1161/01.res.14.3.228 14133949

[B45] Peng Y. J. Overholt J. L. Kline D. Kumar G. K. Prabhakar N. R. (2003). Induction of sensory long-term facilitation in the carotid body by intermittent hypoxia: Implications for recurrent apneas. Proc. Natl. Acad. Sci. U. S. A. 100 (17), 10073–10078. 10.1073/pnas.1734109100 12907705 PMC187770

[B46] Perrini S. Cignarelli A. Quaranta V. N. Falcone V. A. Kounaki S. Porro S. (2017). Correction of intermittent hypoxia reduces inflammation in obese subjects with obstructive sleep apnea. JCI Insight 2 (17), e94379. 10.1172/jci.insight.94379 28878129 PMC5621882

[B47] Philippi N. R. Bird C. E. Marcus N. J. Olson E. B. Chesler N. C. Morgan B. J. (2010). Time course of intermittent hypoxia-induced impairments in resistance artery structure and function. Respir. Physiol. Neurobiol. 170 (2), 157–163. 10.1016/j.resp.2009.12.003 19969108 PMC2821713

[B48] Prabhakar N. R. Kumar G. K. Nanduri J. Semenza G. L. (2007). ROS signaling in systemic and cellular responses to chronic intermittent hypoxia. Antioxid. Redox Signal 9 (9), 1397–1403. 10.1089/ars.2007.1732 17627465

[B49] Prabhakar N. R. Peng Y. J. Nanduri J. (2023). Carotid body hypersensitivity in intermittent hypoxia and obtructive sleep apnoea. J. Physiol. Epub ahead of print. PMID: 37029496. 10.1113/JP284111 37029496

[B50] Puckrin R. Iqbal S. Zidulka A. Vasilevsky M. Barre P. (2015). Renoprotective effects of continuous positive airway pressure in chronic kidney disease patients with sleep apnea. Int. Urol. Nephrol. 47 (11), 1839–1845. 10.1007/s11255-015-1113-y 26424500

[B51] Pügge C. Mediratta J. Marcus N. J. Schultz H. D. Schiller A. M. Zucker I. H. (2016). Exercise training normalizes renal blood flow responses to acute hypoxia in experimental heart failure: Role of the α_1_-adrenergic receptor. J. Appl. Physiol. (1985) 120 (3), 334–343. 10.1152/japplphysiol.00320.2015 26607245 PMC4740500

[B52] Ratliff B. B. Abdulmahdi W. Pawar R. Wolin M. S. (2016). Oxidant mechanisms in renal injury and disease. Antioxid. Redox Signal 25 (3), 119–146. 10.1089/ars.2016.6665 26906267 PMC4948213

[B53] Rey S. Del Rio R. Alcayaga J. Iturriaga R. (2004). Chronic intermittent hypoxia enhances cat chemosensory and ventilatory responses to hypoxia. J. Physiol. 560 (2), 577–586. 10.1113/jphysiol.2004.072033 15319419 PMC1665248

[B54] Roy A. Farnham M. M. J. Derakhshan F. Pilowsky P. M. Wilson R. J. A. (2018). Acute intermittent hypoxia with concurrent hypercapnia evokes P2X and TRPV1 receptor-dependent sensory long-term facilitation in naïve carotid bodies. J. Physiol. 596 (15), 3149–3169. 10.1113/JP275001 29159869 PMC6068228

[B55] Schock-Kusch D. Sadick M. Henninger N. Kraenzlin B. Claus G. Kloetzer H. M. (2009). Transcutaneous measurement of glomerular filtration rate using FITC-sinistrin in rats. Nephrol. Dial. Transpl. 24 (10), 2997–3001. 10.1093/ndt/gfp225 19461009

[B56] Schock-Kusch D. Xie Q. Shulhevich Y. Hesser J. Stsepankou D. Sadick M. (2011). Transcutaneous assessment of renal function in conscious rats with a device for measuring FITC-sinistrin disappearance curves. Kidney Int. 79 (11), 1254–1258. 10.1038/ki.2011.31 21368744

[B57] Sun W. Yin X. Wang Y. Tan Y. Cai L. Wang B. (2012). Intermittent hypoxia-induced renal antioxidants and oxidative damage in male mice: Hormetic dose response. Dose Response 11 (3), 385–400. 10.2203/dose-response.12-027.Cai 23983666 PMC3748850

[B58] Unnikrishnan D. Jun J. Polotsky V. (2015). Inflammation in sleep apnea: An update. Rev. Endocr. Metab. Disord. 16 (1), 25–34. 10.1007/s11154-014-9304-x 25502450 PMC4346503

[B59] Walker L. A. Gellai M. Valtin H. (1986). Renal response to pentobarbital anesthesia in rats: Effect of interrupting the renin-angiotensin system. J. Pharmacol. Exp. Ther. 236 (3), 7–8. 10.1097/00132586-198702000-00008 3512818

[B60] Wang B. Li Z. L. Zhang Y. L. Wen Y. Gao Y. M. Liu B. C. (2022). Hypoxia and chronic kidney disease. EBioMedicine 77, 103942. 10.1016/j.ebiom.2022.103942 35290825 PMC8921539

[B61] Zalucky A. A. Nicholl D. D. Hanly P. J. Poulin M. J. Turin T. C. Walji S. (2015). Nocturnal hypoxemia severity and renin-angiotensin system activity in obstructive sleep apnea. Am. J. Respir. Crit. Care Med. 192 (7), 873–880. 10.1164/rccm.201502-0383oc 26102156

